# Hysterectomy A Comprehensive Surgical Approach

**DOI:** 10.4274/jtgga.2017.0097

**Published:** 2017-12-15

**Authors:** İbrahim Alkatout, Liselotte Mettler

**Affiliations:** 1 Department of Gynecology and Obstetrics, Kiel School of Gynecological Endoscopy, University Hospitals Schleswig-Holstein, Kiel, Germany

**Keywords:** Hysterectomy, laparoscopy, robotic surgery, vaginal hysterectomy, abdominal hysterectomy

## Abstract

This book presents a step-by-step surgical description of vaginal hysterectomy, abdominal hysterectomy, conventional laparoscopic hysterectomy, and robotic-assisted hysterectomy. It brings into balance theoretical background, clinical experience, and scientific findings in a readily comprehensible form with numerous illustrations and tables. The book contains a large proportion of interdisciplinary aspects that make a substantial contribution to meeting the growing requirements of interdisciplinary medical treatment. It offers related disciplines the opportunity to describe areas of common overlap and how these can be treated.

Verschiedene chirurgische Verfahren einschließlich vaginaler Hysterektomie, abdominaler Hysterektomie, konservativer laparoskopischer Hysterektomie und roboterassistierter Hysterektomie werden in diesem englischsprachigen Lehrbuch schrittweise beschrieben. In ausgewogener Art und Weise werden theoretische Hintergründe, klinische Erfahrungen und wissenschaftliche Erkenntnisse in leicht verständlicher Form mit zahlreichen Abbildungen und Tabellen dargestellt. Indem es verschiedene interdisziplinäre Aspekte behandelt, wird das Buch den wachsenden Anforderungen der interdisziplinären medizinischen Therapie gerecht. Benachbarte medizinische Fachbereiche kommen zu Wort und haben die Möglichkeit, überlappende Krankheitsbilder und deren Behandlung zu erläutern.

## INTRODUCTION

This comprehensive surgical approach to hysterectomy rests on the pillars erected by the great masters in gynecology and obstetrics. The first hysterectomy was performed as a vaginal hysterectomy and dates back to ancient times. The procedure was performed in the time of Soranus of Ephesus, 120 years after the birth of Christ. There were many reports of its use in the Middle Ages, nearly always for the extirpation of an inverted uterus, and the patients rarely survived. Hysterectomy became safer with the introduction of anesthesia, antibiotics and antisepsis, blood transfusions, and intravenous therapy. Apart from the transverse abdominal incision introduced by Johannes Pfannenstiel in the 1900s, there was little advance in hysterectomy techniques until the advent of endoscopic surgery and the performance of the first laparoscopic hysterectomy by Kurt Semm in Kiel in 1984, and Harry Reich in Kingston, Pennsylvania in 1988 (1). Stimulated by technical advances, the first hysterectomy with a robotic surgical system was performed after Food and Drug Administration approval in 2005 (2).

In this age of global communication, it seems more than appropriate to publish a specialist surgical book on hysterectomy that features leading surgeons, researchers and teachers from around the globe as contributing authors. With the assistance of over 200 multi-disciplinary authors, the editors have been able to compile a book that meets the requirements of a broad base of readers from beginners to specialists in gynecology. The book at hand also addresses specialists in other surgical fields, such as urologists, visceral surgeons, pathologists, radiologists, anesthesiologists, and conservative gynecologists, dealing with the interdisciplinary challenges associated with hysterectomy.

Although there is a flood of literature on this topic, this comprehensive approach to hysterectomy is unique in that it includes the historical background of the leading surgical techniques of the present day and the future perspectives as seen by the leading, contemporary, multi-disciplinary authors.

## OUTLINE OF THE BOOK CONTENT

The main part of the book provides a balance of theoretical background, broad clinical experience, and scientific findings in a readily comprehensible and well-described manner, enriched with extensive illustrations and tables. For the beginner, this masterpiece serves as a reliable companion, providing background information and assistance for all procedures associated with hysterectomy. This includes abdominal, vaginal, conventional laparoscopic, and robotic-assisted surgical procedures for benign and malignant indications. Experienced surgeons in particular will be able to broaden their spectrum and learn experimental and innovative surgical approaches because this textbook is unique in offering traditional, up-to-date, and innovative surgical methods for hysterectomy. The associated medical fields, such as general surgery, urology, pathology, anesthesiology, radiology, and general /internal medicine, will benefit from the structured organization of the book and the integration of all interdisciplinary aspects.

After beginning with the historical background, there follows a section on topographic anatomy for hysterectomy procedures. This section was written by a renowned clinical anatomist and provides new insights into the female anatomy. The next section comprises five chapters dealing with imaging and diagnostics, followed by a section examining extended, and often controversial, aspects regarding indications and contraindications. Even the often underrepresented aspects of communication and training have a firm place in the book (3). Further topics covered include macroscopic and microscopic pathology, the involvement of antithrombotic therapy, and anesthesiology.

The main aspects of the book deal with the practical performance of hysterectomy with conventional laparoscopic, robotic-assisted, abdominal, and vaginal surgical techniques. In addition to all types of hysterectomies for benign and malignant indications, concomitant procedures such as urogynecologic procedures, lymphadenectomy, and omentectomy are featured in the book.

The book has several unique features. The historical chapters were written by world-renowned and respected contemporary witnesses of pioneering surgical developments (4). The imaging of the anatomic and radiologic chapters is a brilliant realization of current requirements. The chapter on sarcoma and morcellation covers an on-going, far-reaching, health-related policy debate. Innovative and reproducible surgical techniques regarding embryologic resection margins in oncologic cases are presented in different approaches. The initial abdominal approach is compared with the laparoscopic and robotic-assisted surgical approach; such a comprehensive compilation of all surgical techniques has never before been collected together in one book (5). Finally, surgical procedures that are on the verge of becoming lost are reexamined and improved in a brilliant and up-to-date fashion (6).

## EVALUATION OF THE BOOK

The book examines many interdisciplinary aspects and the editors believe it will make a substantial contribution to meeting the growing requirements of interdisciplinary medical treatment. It offers related disciplines the opportunity to describe the areas of common overlap and how these can be treated.

The wide range of contents developed in the course of the conception of the book. Extended hysterectomy procedures cannot be separated from pre- and postsurgical aspects or from procedures on the internal genital organs or those involving the anatomic and functionally-relevant surrounding area. This book gives access to the most advanced treatment concepts of our time. Furthermore, the eBook version meets global demands of unlimited exchange and gives access to the worldwide community interested in this topic. This text book is an important addition to the literature on hysterectomy techniques, accessible to gynecologists worldwide, and thereby contributes towards the global improvement of healthcare for women.
